# Effect of PSI‐697, a Novel P‐Selectin Inhibitor, on Platelet–Monocyte Aggregate Formation in Humans

**DOI:** 10.1161/JAHA.112.006007

**Published:** 2013-02-22

**Authors:** Alan G. Japp, Raj Chelliah, Laura Tattersall, Ninian N. Lang, Xu Meng, Kathleen Weisel, Arie Katz, David Burt, Keith A. A. Fox, Giora Z. Feuerstein, Thomas M. Connolly, David E. Newby

**Affiliations:** 1 Edinburgh Heart Centre, Royal Infirmary of Edinburgh, 51 Little France Crescent, Edinburgh, UK (A.G.J.); 2 British Heart Foundation/University Centre for Cardiovascular Science, University of Edinburgh, 49 Little France Crescent, Edinburgh, UK (R.C., L.T., N.N.L., K.A.F., D.E.N.); 3 Wyeth Research, Collegeville, PA (X.M., K.W., A.K., D.B., G.Z.F., T.M.C.)

**Keywords:** platelets, P‐selectin, thrombosis

## Abstract

**Background:**

Platelet activation is central to the pathogenesis of acute coronary syndromes. Surface expression of P‐selectin on activated platelets induces formation of platelet–monocyte aggregates and promotes vascular inflammation and thrombosis. P‐selectin antagonism may represent a novel therapeutic strategy in vascular disease. We aimed to investigate the effects of the novel P‐selectin antagonist PSI‐697 on platelet–monocyte aggregate formation in humans.

**Methods and Results:**

In a double‐blind, randomized, placebo‐controlled crossover study, healthy smokers were randomized to receive either oral PSI‐697 600 mg or matched placebo. The sequence of treatment was also randomized, with all subjects receiving both PSI‐697 and placebo. Platelet–monocyte aggregates were measured by flow cytometry at 4 and 24 hours in the presence and absence of thrombin receptor‐activating peptide (TRAP; 0.1 to 1.0 μm/L). The *ex vivo* addition of TRAP caused a concentration‐dependent increase in platelet–monocyte aggregates from 8.2% to 94.8% (*P*<0.001). At 4 and 24 hours, plasma concentrations of PSI‐697 increased to 1906 and 83 ng/mL, respectively (*P*<0.001). PSI‐697 had no demonstrable effect on either stimulated or unstimulated platelet–monocyte aggregates at 4 or 24 hours (*P*>0.05). P‐selectin‐blocking antibody (CLB‐Thromb6), but not PSI‐697, inhibited both stimulated and unstimulated platelet–monocyte aggregate formation in vitro (*P*<0.001).

**Conclusions:**

The novel small‐molecule P‐selectin antagonist PSI‐697 did not inhibit basal or stimulated platelet–monocyte aggregate formation in humans at the dose tested. Its clinical efficacy remains to be established.

**Clinical Trial Registration:**

URL: http://EudraCT.ema.europa.eu Unique identifier: 2007‐005695‐14.

## Introduction

P‐selectin is a cell surface adhesion molecule that has a central role in mediating interactions between platelets and other cell types, such as leukocytes and the endothelium.^1^ It is the largest of the selectins, with a mass of 140 kDa, and is stored in the α granules of unstimulated platelets and Weibel–Palade bodies of endothelial cells. When P‐selectin is expressed on activated platelets and endothelial cells, interaction with its primary ligand, P‐selectin glycoprotein ligand type 1 (PSGL‐1), mediates the initial tethering and rolling process that precedes leukocyte transmigration through the vessel wall.^2–3^ There also appears to be a P‐selectin‐dependent pathway of fibrin formation during coagulation; hence, both P‐selectin and PSGL‐1 appear to contribute to thrombus formation and propagation.^4^

Modern flow‐cytometric techniques allow platelets to be analyzed in their physiological environment of whole blood with minimal sample manipulation.^5^ Surface expression of P‐selectin is a marker for platelet activation, and elevated levels have been identified in a number of conditions including cigarette smoking,^6^ diabetes mellitus,^7^ hypertension,^8^ and acute coronary syndromes.^9^ In addition, circulating platelet–monocyte aggregates form by the binding of activated platelets to leukocytes through a P‐selectin‐dependent mechanism.^9–10^ This can be readily measured by flow cytometry and has emerged as a highly sensitive marker of platelet activation.^11^ The adhesion of activated platelets to monocytes also has important functional consequences and can induce the expression of cytokines, chemokines, adhesion molecules, and tissue factor.^12–13^ Consistent with these biological effects, circulating platelet–monocyte aggregates appear to promote atherosclerotic lesion formation^14^ and are increased in patients with stable coronary heart disease^15^ and acute coronary syndromes.^9^

The therapeutic potential of P‐selectin antagonism has been demonstrated by the application of specific monoclonal antibodies that block P‐selectin and PSGL‐1 and are associated with reduced levels of platelet activation.^9^ Indeed, P‐selectin‐blocking antibodies reduced arterial thrombosis, reperfusion injury, and infarct size in mice.^16^ Recently small‐molecule antagonists have been developed that interact with P‐selectin and inhibit its function. PSI‐697 is a novel small‐molecule selective P‐selectin antagonist that reduces both arterial and venous thrombosis in animal models.^17–20^

The aim of the present study was to assess the effects of PSI‐697 on platelet–monocyte aggregate formation in healthy smokers and nonsmokers.

## Methods

### Subjects

In all, 25 smoking and 6 nonsmoking healthy volunteers were recruited into the study following screening. Before study commencement, all subjects had a normal electrocardiogram and normal baseline measures of clinical hematology and biochemistry variables including hemoglobin, platelet count, renal function, glucose, and coagulation. Exclusion criteria included intercurrent infective illness, diabetes mellitus, hypertension, renal and hepatic impairment, asthma, bleeding disorder, alcohol or drug misuse, recent surgical procedures, and the use of regular medications.

The study was performed with the approval of the research ethics committee in accordance with the Declaration of Helsinki and with the written informed consent of all volunteers.

### Drugs

PSI‐697 (2‐[4‐chlorobenzyl]‐3‐hydroxy‐7,8,9,10‐tetrahydrobenzo[h] quinoline‐4‐carboxylic acid) is a selective antagonist of P‐selectin.^17,20^ For in vitro studies, PSI‐697 was dissolved in polyethylene glycol (molecular weight 400) and diluted (1:10) in a saline vehicle. Based on previous pharmacokinetic data from ascending‐dose clinical studies, a single oral dose of 600 mg of PSI‐697 was selected to achieve a plasma concentration of ≈1700 ng/mL at 4 hours and 170 ng/mL at 24 hours. In a canine thrombosis model, PSI‐697 at a plasma concentration of 835 ng/mL reduced platelet–monocyte aggregate formation (unpublished results).

### Flow Cytometry

Peripheral venous blood was drawn from a large antecubital vein with a 19‐gauge needle and anticoagulated with the direct thrombin inhibitor d‐phenylalanine‐l‐prolyl‐l‐arginine chloromethyl ketone (PPACK, 75 μmol/L; Cambridge Biosciences, UK).^21^ Immediately following venesection, aliquots of whole blood (500 μL) were incubated for 5 minutes in the presence or absence of thrombin receptor‐activating peptide (TRAP; 0.1, 0.3, and 1.0 μmol/L) to induce platelet activation. Sample aliquots (60 μL) were then incubated with anti‐CD14‐PE, anti‐CD42a‐FITC, and isotype matched controls for 20 minutes at room temperature. Thereafter, samples were fixed and the red cells lysed by the addition of 500 μL of FACS‐Lyse solution. Samples were then stored at 4°C and analyzed on a FACS Calibur flow cytometer (Becton Dickinson) using Cell Quest software as described previously. Samples were run at a medium flow rate until 2500 monocytes were collected. Monocytes were identified by their forward‐ and side‐scatter characteristics as well as by their binding to CD14. CD42a‐positive monocytes were taken to be platelet–monocyte aggregates (PMA) and expressed as a percentage of cells.

### Measurement of Plasma PSI‐697 Concentrations

PSI‐697 concentrations were determined using a validated LC/MS/MS method. Based on a sample volume of 200 μL, the method had a lower limit of quantification of 4.0 ng/mL. The interday coefficients of variation (CVs) and mean bias values for the calibration standards ranged from 3.7% to 7.8% and from −3.9% to 2.6%, respectively. The interassay CV for the quality control samples containing PSI‐697 at 12.0, 350, and 800 ng/mL were 10.3%, 6.2%, and 7.4%, respectively. Mean inaccuracy (bias) values were −2.5%, −5.4%, and −0.4% for 12.0, 350, and 800 ng/mL, respectively.

### Study Design

#### In vitro studies in nonsmokers

Six healthy nonsmokers attended on a single occasion for venipuncture after a minimum 4‐hour fast. Either PSI‐697 (7400 ng/mL; 20 μmol/L), the P‐selectin‐blocking antibody CLB‐Thromb6 (10 μg/mL) or control buffer was immediately added to whole blood before measuring platelet–monocyte aggregates in the presence or absence of TRAP stimulation as described above.

#### Oral administration of PSI‐697 in smokers

Twenty‐five healthy smokers attended on 2 occasions 1 week apart. On each occasion, they were admitted to the Edinburgh Clinical Research Facility for an observed overnight fast of ≥10 hours. A brief physical assessment was performed, with recording of vital signs, 12‐lead electrocardiogram, and laboratory evaluations of blood chemistry, hematology, coagulation, urinalysis, and urine drug screen. Study medication was administered at 8 am and blood drawn at 12 pm and at 8 am the following morning. Heart rate, blood pressure, respiratory rate, and body temperature were observed for 6 hours post dosing. Subjects were free to smoke throughout the study period and were encouraged to smoke at least 2 cigarettes on the morning of study, but not more than 30 minutes before dosing.

### Statistical Analysis

In vitro study data are presented as mean±standard error of the mean (SEM) and were analyzed with 2‐way analysis of variance (ANOVA) with repeated measures. For the in vivo study, data are presented as mean±standard deviation (SD). In this study, the change from baseline values for PMA were analyzed using a mixed‐effects analysis of covariance (ANCOVA) that accounts for repeated measures taken within a 2‐period crossover design. Baseline values were included in the model as a covariate. Treatment (PSI‐697 or placebo), time, period, and sequence as well as the interaction between time and treatment were included in the model as fixed effects. Subject was a random effect in the model. Statistical significance was taken as a 2‐sided *P* value <0.05.

## Results

### In Vitro Studies in Nonsmokers

**Figure 1.**
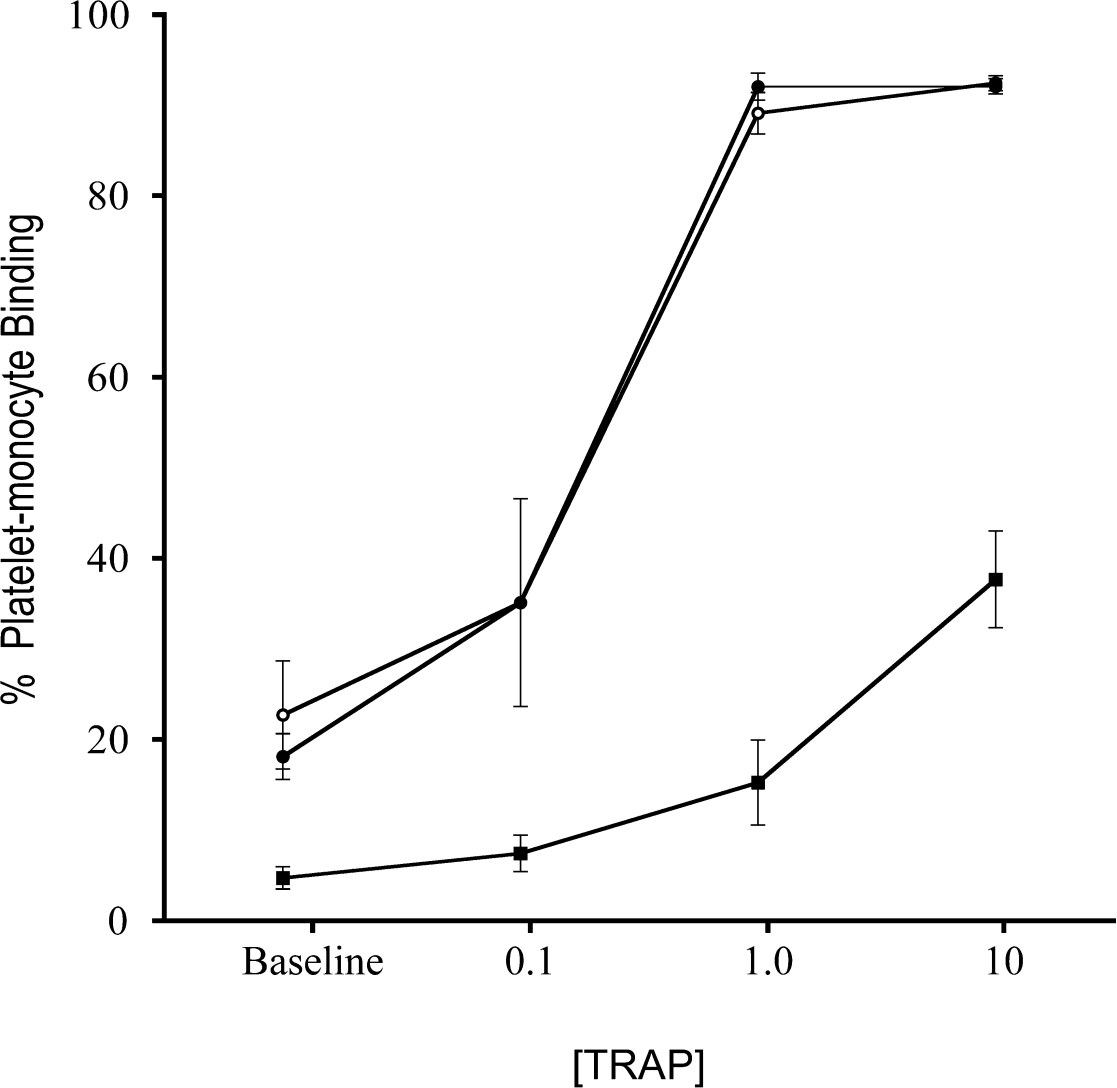
Platelet–monocyte aggregates (%) following thrombin receptor‐activating peptide (TRAP) activation in the presence of saline (open circles), the P‐selectin antagonist PSI‐697 (20 μmol/L [7400 ng/mL]; closed circles), and the P‐selectin‐blocking antibody CLB‐Thomb6 (10 μg/mL; closed square). *P*<0.001 for platelet–monocyte aggregate formation in response to TRAP;* P*<0.001, CLB‐Thromb6 vs placebo; *P*>0.05, PSI‐697 vs placebo.

In vitro analyses were conducted in 6 healthy nonsmoking male and female volunteers aged between 18 and 30 years. The in vitro addition of TRAP produced a concentration‐dependent increase in platelet–monocyte aggregate formation (*P*<0.001; Figure 1). The P‐selectin‐blocking antibody CLB‐Thromb6 markedly inhibited platelet–monocyte aggregate formation in the presence and absence of TRAP (*P*<0.05). In contrast, we observed no difference in either unstimulated or stimulated platelet–monocyte aggregates following the addition of PSI‐697 compared with placebo control.

### Oral Administration of PSI‐697 in Smokers

One hundred twenty‐two smokers were screened to identify 25 volunteers to participate in the study. Twenty‐four of the 25 recruited volunteers completed the study visits; 1 subject declined to participate further after 1 visit for personal reasons. Subjects smoked 18±4 cigarettes daily and were white men aged 34±9 years (range, 18 to 55 years) with a body mass index of 24±3 kg/m^2^.

Following oral administration, mean (±SD) plasma PSI‐697 concentrations rose to 1906±1511 ng/mL at 4 hours and subsequently fell to 83±52 ng/mL at 24 hours. PSI‐697 was well tolerated, with no clinically significant symptoms or changes in hematological, biochemical, or electrocardiographic variables.

**Figure 2.**
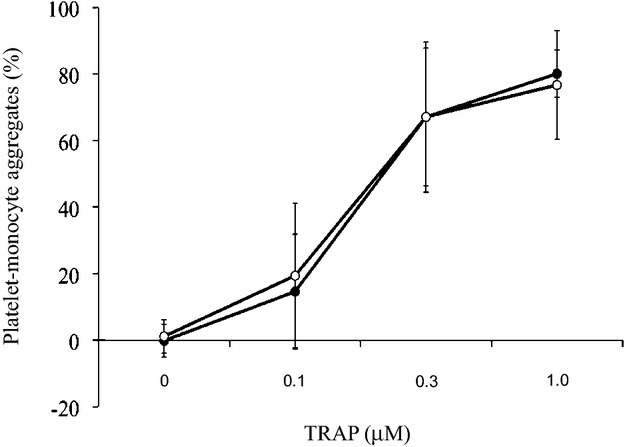
Platelet–monocyte aggregates (%) following thrombin receptor‐activating peptide (TRAP) activation 4 hours following oral administration of the P‐selectin antagonist PSI‐697 (600 mg; closed circles) or matched placebo (open circles). *P*<0.001 for platelet–monocyte aggregate formation in response to TRAP;* P*>0.05, PSI‐697 vs placebo.

**Figure 3.**
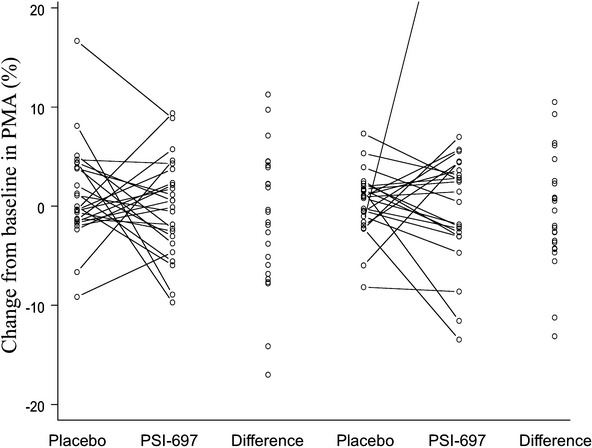
Individual values for absolute change from baseline in platelet–monocyte aggregates 4 hours (left panel) and 24 hours (right panel) after oral administration of the P‐selectin antagonist PSI‐697 or matched placebo.

Platelet–monocyte aggregates increased in a concentration‐dependent manner with the addition of *ex vivo* TRAP (Figure 2). Platelet–monocyte aggregate measurements ranged from 3.7% to 41.4% for unstimulated samples and from 8.2% to 94.8% for stimulated samples. There was no difference in stimulated and unstimulated platelet–monocyte aggregates between placebo and PSI‐697 (*P*>0.05; Figure 3). At 4 hours, there was a difference estimate of −1.2% (95% confidence interval [CI], −3.6% to 1.1%; *P*=0.3013) of platelet–monocyte aggregation from baseline. At 24 hours, there was a difference estimate of 0.9% (90% CI, −2.9% to 4.7%; *P*=0.6304) of platelet–monocyte aggregation. Of all of the fixed effects included in our ANCOVA model, only the baseline PMA value was significant (*P*<0.0001).

## Discussion

We have demonstrated that oral administration of the P‐selectin antagonist PSI‐697 was well tolerated and caused no significant adverse effects in otherwise healthy smokers. However, PSI‐697 was unable to block the P‐selectin‐PSGL‐1 binding of activated platelets to monocytes, either in vitro or in vivo, at the dose tested. This suggests a lack of efficacy of PSI‐697 or an inability of this biomarker to assess P‐selectin antagonism in humans.

The therapeutic approach of blocking P‐selectin action has several potentially beneficial effects in the prevention or treatment of cardiovascular disease. P‐selectin enables the tethering and rolling of leukocytes along the vessel wall^2–3^ and promotes the generation of prothrombotic microparticles, leading to the development of thrombosis.^22^ Interruption of this early event in lesion formation may prevent downstream amplification of the inflammatory and thrombotic processes that contribute to atherosclerotic lesion growth, development, and instability.^9^ Moreover, studies have suggested that there is a P‐selectin‐dependent pathway of fibrin formation during coagulation, and hence both P‐selectin and PSGL‐1 contribute to thrombus formation and propagation.^4,23–24,22,25^

We^9^ and others^10–13^ have previously demonstrated that P‐selectin interaction with its primary ligand, PSGL‐1, mediates the majority of platelet–monocyte aggregate formation. Platelet–monocyte aggregate formation is a measure of in vivo platelet activation that is readily undertaken, is reproducible, and can be augmented by the addition of exogenous agonists.^21^ Therefore, this would seem to be the ideal biomarker to determine the efficacy of P‐selectin antagonism. Why then did we not see an effect of PSI‐697 on platelet–monocyte aggregates?

Did we achieve sufficient plasma PSI‐697 concentrations following oral dosing? We based the oral dosing on previous phase I pharmacokinetic data in health volunteers. To avoid any interaction with diet that could affect bioavailability, subjects were fasted prior to drug administration. Four hours following oral administration, we achieved the anticipated plasma PSI‐697 concentration of 1906 ng/mL. This concentration should be sufficient to inhibit P‐selectin‐mediated platelet–monocyte aggregate formation, as it was higher than the in vivo concentration that inhibited platelet–monocyte aggregate formation by ≈30% in a canine thrombosis model (unpublished observations). Therefore, we concluded that the absence of a demonstrable effect was unlikely to be a result of inadequate dosing or lack of bioavailability. This could be confirmed in future studies by determination of ligand binding of PSI‐697 at equivalent plasma concentrations.

Are platelet–monocyte aggregates an appropriate biomarker of P‐selectin activity? To confirm our current model, we measured in vitro basal and stimulated platelet–monocyte aggregate formation in the presence and absence of P‐selectin antagonism with either PSI‐697 or a P‐selectin‐blocking antibody. While acknowledging that our study may have been underpowered to detect small differences, the addition of PSI‐697 had no apparent effect on platelet–monocyte aggregation. In contrast, the P‐selectin‐blocking antibody markedly inhibited platelet–monocyte aggregate formation. This reaffirms our previous findings^9^ and those of others^10–13^ that platelet–monocyte aggregate formation is a predominantly P‐selectin‐mediated process. We acknowledge there remains a small but persistent residual proportion of platelet–monocyte aggregates that are formed by P‐selectin‐independent mechanisms.^9^ Blockade of the P‐selectin pathway may upregulate these other mechanisms and allow aggregate formation. However, the P‐selectin‐blocking antibody was able to achieve marked inhibition, and if PSI‐697 was efficacious, then we would have anticipated a demonstrable reduction in aggregate formation. We therefore believe that the biomarker used in this study was appropriate.

Why does PSI‐697 not block the formation of platelet–monocyte aggregates in humans? The present study is the first to assess the effects of oral administration of PSI‐697 on platelet–monocyte aggregates in humans. In several preclinical models, PSI‐697 at concentrations as low as 400 ng/mL has demonstrated favorable reductions in both arterial and venous thrombosis.^17–20^ Oral administration of PSI‐697 to rodents reduced inflammation and thrombus formation that importantly was not associated with prolonged bleeding times.^17,19^ In baboon models of venous thrombosis, PSI‐697 decreased vascular inflammation, modified thrombogenesis, and encouraged recanalization of thrombosed veins without measurable anticoagulation.^18^ Our study did not assess the effects of PSI‐697 on vascular thrombosis and focused on the binding of platelets to monocytes. Thus, there seems to be inconsistency between the antithrombotic effects of PSI‐697 and its ability to inhibit platelet–monocyte aggregates. Indeed, we have demonstrated that PSI‐697 reduces *ex vivo* thrombus formation in humans at concentrations achieved in the current study.^26^ Using the Badimon *ex vivo* model of thrombosis, we have shown that, under dynamic flow conditions at both high and low shear stress, PSI‐697 caused a reduction in thrombus formation.

How do we account for the discrepancy between thrombosis and platelet–monocyte aggregate data? This is difficult to reconcile but could include an off‐target effect of PSI‐697 that has an antithrombotic action mediated through a non‐P‐selectin pathway. Alternatively, the interaction of PSI‐697 with P‐selectin may be incomplete in certain settings. There are 2 ligand recognition sites on P‐selectin: sialyl Lewis x and PSGL‐1 core protein.^27^ Sialyl Lewis x is a carbohydrate on the cell surface attached to an O‐glycan and plays a vital role in cell recognition processes. It is this component that PSI‐697 mimics and causes P‐selectin antagonism. However, it may be that platelet–monocyte aggregate formation does not require binding of both sites and may explain the apparent contradictory findings with the P‐selectin‐blocking antibody. This hypothesis requires further study.

Smoking is associated with accelerated atherosclerotic development,^28^ with an increase in markers of systemic inflammation. We have previously demonstrated that cigarette smoking is associated with increased baseline platelet activation with modest increases in platelet–monocyte aggregates.^29^ In the present study, many of the cigarette smokers had low numbers of platelet–monocyte aggregates, contrasting with our previous findings. We believe that this is likely to reflect the strict inclusion criteria of the present study and that we selected a “healthier” population of smokers than found in our previous study. We do not believe this detracts from our findings because we also assessed TRAP‐induced platelet–monocyte aggregates and achieved very high levels of aggregate formation in vitro. Nonetheless, further studies examining the potential effects of PSI‐697 with agonists other than TRAP and in populations with higher baseline levels of platelet–monocyte aggregates, such as patients with diabetes mellitus or established vascular disease, would provide useful confirmation of our findings.

## Conclusions

The novel small‐molecule P‐selectin antagonist PSI‐697 did not inhibit basal or stimulated platelet–monocyte aggregate formation in humans at the dose tested. Its clinical efficacy remains to be established.
